# Decrease in Overall Vaccine Hesitancy in College Students during the COVID-19 Pandemic

**DOI:** 10.3390/vaccines11071132

**Published:** 2023-06-21

**Authors:** Kendall Pogue, Jessica D. Altman, Abigail A. Lee, Dashiell S. Miner, Ty J. Skyles, Ruth J. Bodily, Triston B. Crook, Bryce U. Nielson, Kaitlyn Hinton, Lydia Busacker, Zoe E. Mecham, Agnes M. Rose, Scott Black, Brian D. Poole

**Affiliations:** 1 Department of Microbiology and Molecular Biology, Brigham Young University, Provo, UT 84602, USA; 2 Department of English, University of Utah, Salt Lake City, UT 84112, USA

**Keywords:** vaccine hesitancy, COVID-19, protective equipment

## Abstract

The COVID-19 pandemic changed our world as we know it and continues to be a global problem three years since the pandemic began. Several vaccines were produced, but there was a considerable amount of societal turmoil surrounding them that has affected the way people view not only COVID-19 vaccines but all vaccines. We used a survey to compare how attitudes towards vaccination have changed in college students during the pandemic. An initial survey was administered in 2021, then a follow-up in 2022. Out of 316 respondents who answered the first survey, 192 completed the follow-up. The survey was designed to measure trends in changes to vaccine attitudes since the COVID-19 pandemic began. By comparing the first survey in 2021 and the follow-up, we found that roughly 55% of respondents’ vaccine attitudes did not change, roughly 44% of respondents’ attitudes towards vaccines became more positive, and only about 1% of the respondents’ vaccine attitudes became more negative. Improved view of vaccines was associated with political views and increased trust in medicine and the healthcare system. Worsened opinions of vaccines were associated with a belief that the COVID-19 vaccine affected fertility.

## 1. Introduction

Before the COVID-19 pandemic, the total number of people vaccinated in the world was at an all-time high despite increasing pockets of vaccine resistance. According to the World Health Organization, the number of infants vaccinated annually was 116 million. A total of 86% of all infants had been vaccinated, the most ever recorded [1]. In Immunization Agenda 2030, the World Health Organization published ambitious goals to reduce all yellow fever outbreaks to 0 by 2026, eliminate meningitis epidemics by 2030, and certify poliovirus eradication by 2023 [1]. However, the advent of the COVID-19 pandemic dashed the hopes of any quick improvements in the near future. Health clinics around the world shut down [2,3], mass vaccination campaigns were canceled [2,4], and the COVID-19 vaccine was brought into the international spotlight.

Several factors have been found to affect COVID-19 vaccine hesitancy. While lack of trust, anti-vaccine attitudes, and a need for more information were prominent prior to the COVID-19 vaccine release in the fall of 2020 [5], most concerns about the COVID-19 vaccines centered on efficacy and safety [6,7,8]. One sentiment that initially disturbed the populous was the possibility that the COVID-19 vaccine was rushed [6,9,10]. Many people have vocalized a desire to see further proof and development of the vaccine before they receive it [7,9]. Such concerns may have been ameliorated as people have seen the safety of the vaccine in their lives [11]. A crucial factor in COVID-19 vaccine hesitancy is access to reliable information about the pandemic and vaccines. A recent study examining older adults suggested that increased access to health information is a positive predictive factor of vaccination [9]. Since the public’s access to information regarding the pandemic has improved over time, it is possible that COVID-19 vaccine attitudes have improved [10]. A systematic review of the pandemic showed that COVID-19 vaccine attitudes had improved by 7.4% at the time the survey was conducted (November 2021) from the time the vaccines were introduced [5]. 

Recent studies suggest that primarily obtaining vaccine-related information from social media is negatively correlated with vaccine uptake [12,13]. In addition, variables such as sex, race, age, education level, employment, and income have all been studied as potential determinants of vaccine hesitancy [5,6,10,14,15]. In particular, differences in vaccine uptake between different ethnicities have been shown to be present across the world. In the UK, Black, Pakistani, and Bangladeshi groups had higher rates of vaccine hesitancy across the pandemic compared to the overall population [11]. Similarly in the US, higher vaccine hesitancy was found in participants of color, most notably in Black populations [8,14,16,17,18]. 

Previous studies have noted that this hesitancy may stem from historic oppression [19]. While simultaneously experiencing higher rates of vaccine hesitancy, minority communities have experienced especially high rates of COVID-19 infection, hospitalization, and mortality [20,21,22], creating a higher COVID-19 burden for these communities. Longitudinal studies in the US have also noted that lower education levels and lower socioeconomic status correlate with increased vaccine hesitancy, lower vaccine uptake, and increased hospitalization and mortality rates [8]. During the COVID-19 pandemic, observations were made about how this pandemic has affected the vaccination intentions of individuals for routine vaccinations, not just the COVID-19 vaccine. Several studies found that there has been a decrease in pro-vaccination attitudes during the pandemic [23,24,25]. In particular, there seems to be more parental resistance against routine childhood vaccinations since the COVID-19 vaccine and the community closures [24,25]. This hesitancy can be linked to factors such as distrust in COVID-19 policies, fear of exposure to the virus itself, and misinformation from anti-vaccination sources [24,25,26]. One source found that misinformation is able to spread faster throughout the world than the virus itself can [4]. The spread of untrue statements has created parental anxiety about vaccinating their children [27,28]. These concerns, as well as other factors such as lack of access, have led to decreased uptake of standard childhood vaccines during the COVID-19 pandemic [2]. 

These examples suggest that a myriad of different factors influencing vaccine attitudes have come up over time and that these vary among different demographical groups. Therefore, a longitudinal analysis is needed in order to clarify trends in generalized vaccine hesitancy that has occurred since the pandemic began. We conducted a longitudinal study on college students at two different time points in the pandemic and surveyed vaccine attitudes and other factors that may have impacted their decision to get a vaccine. The first survey was given to students at Brigham Young University and the University of Utah in February 2021 and the follow-up survey was given to the same participants in September 2022. Included in the survey were questions aimed at better understanding the various factors that impacted general vaccine hesitancy in college students over this unprecedented pandemic. While other studies have examined the effect of the COVID-19 pandemic on general vaccine attitudes in various populations, to our knowledge, this is the first US-based longitudinal study assessing this phenomenon specifically in this age group. We hypothesized that experiencing and living through the COVID-19 pandemic caused an increase in positive attitudes about vaccines in general in college students in comparison with their vaccine attitudes at the beginning of the COVID-19 pandemic.

## 2. Materials and Methods

### 2.1. Study Outcomes

The objective of this study was to determine the effect of the COVID-19 pandemic on attitudes towards vaccination in general. The primary survey item used to determine this objective was “How has your experience with the global COVID-19 pandemic affected your opinion on vaccines in general?”

The secondary objective of the study was to understand the factors that contributed to a change in opinion on vaccines in general. Several variables were hypothesized to have an impact on participants’ vaccine attitudes. These included trust in the pharmaceutical industry, trust in healthcare, trust in medicine, trust in government, perceived impact of the COVID-19 pandemic, political views, news and media sources, trust in public measures, and perceived fertility issues with the COVID-19 vaccines.

### 2.2. Measures

#### 2.2.1. Study Design

In February 2021, our team designed and administered a survey with the goal of measuring the impact of COVID-19, knowledge and attitudes regarding the disease, and many factors regarding vaccinations and intent to vaccinate. Demographic factors such as race, age, sex, and income were also measured. In September 2022, a second survey was administered to the respondents of the original survey in order to identify any changes in their attitudes about vaccines over the course of the COVID-19 pandemic. The second survey also included several new variables that became apparent due to the COVID-19 pandemic and were hypothesized to have an impact on vaccine attitudes. Due to the inclusion of both common items and additional items in the 2022 survey, this study has longitudinal elements and cross-sectional elements. In the original survey, 316 responses were recorded, and 192 (61%) of the original respondents completed the second survey. Anyone who responded to the first survey but not the second survey was not included in this follow-up study. The responses from 2022 were compared to the responses from the 2021 survey. The project was approved by the Brigham Young University Institutional Review Board (Approval number IRB2020-342). 

#### 2.2.2. Development and Validation of Instrument

Survey questions were based on our previous work, specifically [9]. These items were examined using confirmatory factor analysis for validity and reliability. Appropriate items from the survey in this prior study were copied into the present study. New questions were written and checked for face validity and comprehension by both a professor and undergraduate students.

#### 2.2.3. Sample Size Estimation

Power analysis for an alpha of 0.05 and a beta of 0.2 was performed after the initial survey. The analysis was based on the question: “How has your experience with the global COVID-19 pandemic affected your opinion on vaccines in general?” With a mean of 2.54 and a standard deviation of 0.8, the power analysis indicated a need for 198 subjects in the first study and 99 in the second. This included an estimated 50% dropout between surveys. 

### 2.3. Sampling

#### 2.3.1. Study Site

Both surveys were administered by Qualtrics (Provo, UT, USA) to students in General Education classes at Brigham Young University in Provo, UT, USA, and the University of Utah, in Salt Lake City, UT, USA.

#### 2.3.2. Sampling Technique

General education teachers at Brigham Young University and the University of Utah sent a recruiting email to members of their classes to participate in the survey. Compensation was provided in the form of extra credit. Participants provided consent to be contacted for a follow-up survey upon participation in the initial survey.

The survey was not anonymous because of the inclusion of email addresses for future follow-up. However, the responses were de-identified by coding the responses, then removing the email addresses. The participants knew that the survey would not be anonymous, as they were asked to provide their email addresses. However, they were assured that their professors would not have access to their email addresses. The same participants filled out the second survey because they were individually invited using their email addresses. These were compared to the codes from the first survey to match respondents for the first and second surveys. 

**Figure 1:**
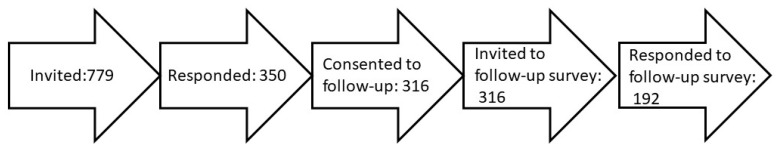
Flow diagram of survey participants. A total of 779 students were invited to participate. Out of those, 316 finished the survey and consented to receive a follow-up survey (40.6%). These 316 were invited to participate in the follow-up survey. Out of these, 192 completed the follow-up survey (60.1%).

Participants who completed the first survey were recruited to participate in the second survey. Emails were sent to these subjects at the emails they provided in the response to the initial survey. Both surveys were recruited via email notification. Survey flow is shown in Figure 1. Both surveys are available in the Supplemental Materials (Supplementary Files S1 and S2).

### 2.4. Data Collection and Handling

Survey participants were recruited by email as described above. The emails included a link to the survey which was administered by Qualtrics. Qualtrics also handled data acquisition and storage.

### 2.5. Statistical Analysis

Wilcoxon signed-rank tests were used to detect significant changes in individual items between the original and follow-up surveys. Pearson’s correlation analysis was used to perform univariate analyses on the follow-up survey. Correlational analyses were done by comparing question 4.1 on the survey, which is “How has your experience with the COVID-19 global pandemic affected your opinion on vaccinations in general (not including the COVID-19 vaccine)?” to the relevant other survey questions. Chi-Square analysis was performed to determine any dependency of categorical variables on the answers for question 4.1. 

Multivariate analysis was performed using a stepwise ordinal logistic model. The stepwise regression compared each of nine items to question 4.1 on the survey to determine their significance.

## 3. Results

### 3.1. Samples and Demographics

This study surveyed 192 individuals who responded to both the initial and the follow-up surveys. The demographic information of the respondents is presented in Table 1. Since this study was performed at universities in Utah, the predominant age of the respondents is 18–25, and racial diversity is low. 

### 3.2. The Influence of COVID-19 on General Vaccine Attitudes

**Figure 2:**
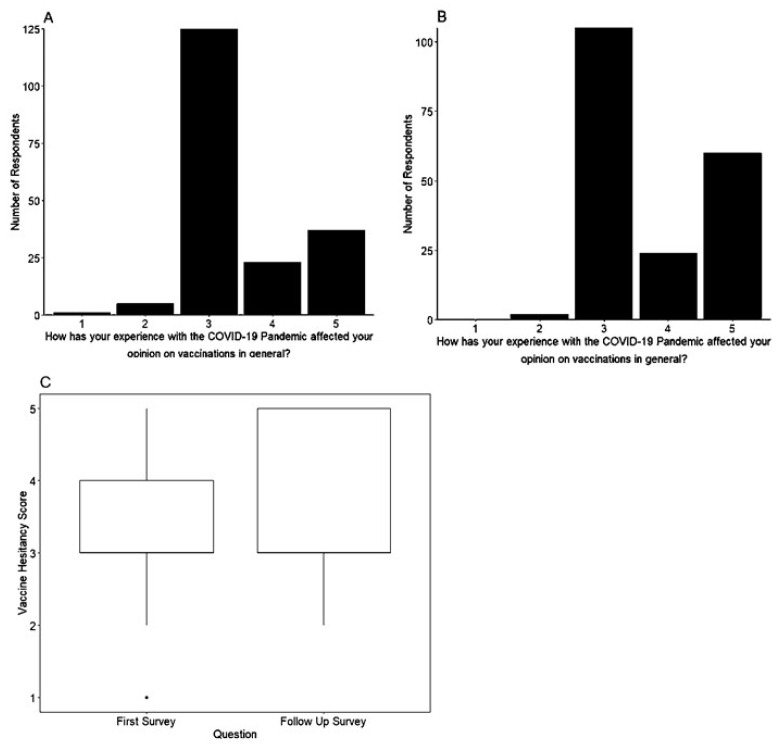
The COVID-19 pandemic improved vaccine attitudes in the study population. (**A**) The distribution of answers to the following question in the initial survey (February 2021) “How has your experience with the COVID-19 Pandemic affected your opinion on vaccinations in general?” is shown. Answers range from “I am much more likely to vaccinate myself/my children” to “I am much less likely to vaccinate myself/my children”. Higher numbers indicate greater favorability and lower numbers indicate lower favorability. (**B**) Responses to the same question in the follow-up survey Jan 2022. (**C**) Comparison of the responses between the first and second surveys. Responses showed a significantly more favorable impact of the COVID-19 vaccine pandemic on overall vaccination intent in the later survey (Wilcoxon signed-rank test, *p* < 0.001).

The question “How has your experience with the COVID-19 global pandemic affected your opinion on vaccinations in general (not including the COVID-19 vaccine)?” was asked on both the initial and follow-up surveys (Figure 2). When looking at changes in vaccine attitudes due to the COVID-19 pandemic between the respondents’ paired answers, we saw that in the first survey, a total of 64.92% of respondents reported no change in opinions on vaccinations. By the second survey, a total of 55.21% of the study subjects reported no change in opinions on vaccinations in response to the same question. 

In the initial survey, 31.94% of the paired respondents said they were “much more likely to vaccinate myself/my children” (19.37%) or “more likely to vaccinate myself/my children” (12.57%). In the follow-up survey, this number increased, with a total of 43.75% of the study subjects saying they were “much more likely to vaccinate myself/my children” (31.25%) or “more likely to vaccinate myself/my children” (12.50%). 

In the initial survey, 3.14% of study subjects said they would be “less likely to vaccinate myself/my children” (2.62%) or “much less likely to vaccinate myself/my children” (0.52%). 

In the follow-up survey, only 1.04% of study subjects said they would be “less likely to vaccinate myself/my children”, and no respondents said that they are “much less likely to vaccinate myself/my children”. The change in intent to vaccinate between the first and second surveys was statistically significant (*p* < 0.001) (Figure 2). The respondents had an increased positive attitude towards vaccines after experiencing the COVID-19 pandemic. 

### 3.3. Effect of the Pandemic on the Perception of Vaccine Side Effects

**Figure 3:**
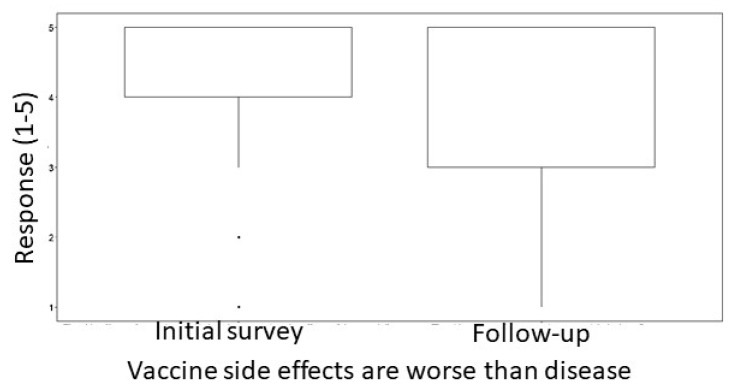
Side effects became a larger concern during the COVID-19 pandemic. Participants were asked how much they agreed with the statement “Side effects of vaccines are worse than the disease”. Responses could be from 1-Strongly disagree to 5-Strongly agree. Wilcoxon signed-rank test showed a statistically significant difference between the two groups, with a shift of greater agreement with the idea that a vaccine has side effects that are worse than the disease (*p* < 0.00001).

In both surveys, the respondents were asked to rank how they felt about various statements regarding vaccines. There were also items that examined the respondents’ general knowledge about vaccines and the COVID-19 pandemic. In the initial survey, a substantial number of respondents (although not a majority) worried that the side effects of a potential vaccine would be worse than the disease itself. When presented with the statement “The side effects of the vaccine are likely to be worse than COVID-19 itself”, 19.81% selected “strongly agree” and 19.50% selected “Somewhat agree”. In total, 14.86% selected “strongly disagree”, 16.72% selected “somewhat disagree”, and 29.10% neither agreed nor disagreed. However, in the follow-up survey, a significant amount of people agreed with this statement less. In the initial survey, the mean response of individuals was 4.51. However, in the follow-up survey, the mean response of individuals was 3.81. A Wilcoxon signed-rank test showed statistical significance between these groups (Figure 3). 

### 3.4. Effect of the Pandemic on Mask Effectiveness 

**Figure 4:**
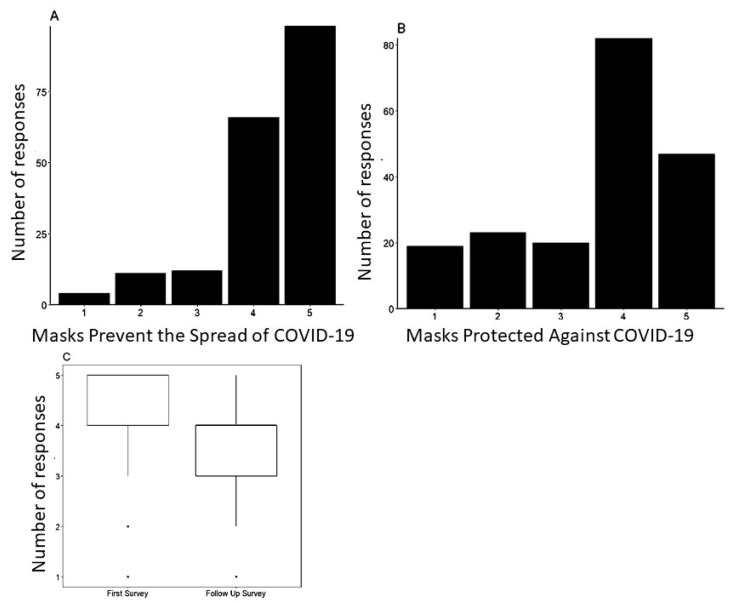
Pandemic experience decreased trust in mask use. Participants were asked to respond to the statement “When worn correctly, masks prevent the spread of COVID-19” in the initial survey (**A**) and “Masks were effective in protecting against COVID-19” in the second survey (**B**). Responses could be from 1-Strongly disagree to 5-Strongly agree in both cases. (**C**) Responses were compared between the first survey and the second survey. There was a statistically significant difference between the first and second surveys, with more people disagreeing with the idea that masks helped prevent the spread of COVID-19 in the follow-up survey (*p* < 0.00001, Wilcoxon signed-rank test).

Participants were asked about the effectiveness of masks in protecting against COVID-19 in the initial and follow-up surveys. Participants answered on a five-point scale, from a possibility of ‘Strongly disagree’ to ‘Strongly agree’. While numbers indicating trust in masks were high in the first survey, mean values dropped from 4.272 in the initial study to 3.602 in the follow-up study. Results showed a statistically significant difference between students in the first and follow-up survey, showing that fewer people believed in the effectiveness of masks as the pandemic progressed (Figure 4).

### 3.5. The Influence of COVID-19 on Trust in Pharmaceuticals, Medicine, and Government

**Figure 5:**
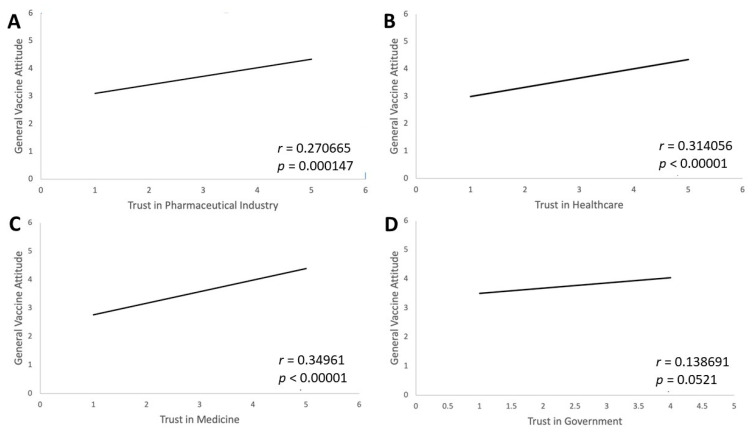
Trust in vaccination sources correlates with general vaccine attitudes. Four follow-up survey questions measured change in trust in institutions during the pandemic. These were (**A**) “During the pandemic, my trust in the pharmaceutical industry has…”, (**B**) “During the pandemic, my trust in healthcare has…”, (**C**) “During the pandemic, my trust in medicine has…”, and (**D**) “During the pandemic, my trust in government has…” with answer choices from “Decreased greatly” to “Increased greatly”. Responses were compared to the item “How has your experience with the COVID-19 global pandemic affected your opinion on vaccinations in general (not including the COVID-19 vaccine)?” There was a positive correlation between increased trust in the pharmaceutical industry (*p* = 0.000147), healthcare (*p* < 0.0001) and medicine (*p* = 0.000147) and increased general vaccine attitudes. Changed trust in the government and changed general vaccine attitudes were not found to have a significant correlation (*p* = 0.055).

Trust in vaccination sources on individuals’ vaccination attitudes in general was examined (Figure 5). A positive correlation was found between increased trust in the pharmaceutical industry and higher intent to vaccinate in general (r = 0.270665, *p* < 0.001) (Figure 5A). Similarly, there is a strong positive correlation between higher trust in healthcare (r = 0.314056, *p* < 0.001) and higher trust in medicine (r = 0.34961, *p* < 0.001) when compared with vaccination attitudes in general (Figure 5B,C). A significant correlation was not found between trust in the government and general vaccination attitudes (r = 0.138691, *p* = 0.05521) (Figure 5D). Those who had increased trust in trust in the pharmaceutical industry, healthcare, and medicine as a result of the COVID-19 pandemic were also more likely to say that the pandemic had a positive impact on their intent to vaccinate in general. An individual’s trust in government, however, does not seem to be significantly correlated with their attitude towards vaccination in general. 

### 3.6. Political Attitudes and Intent to Vaccinate

**Figure 6:**
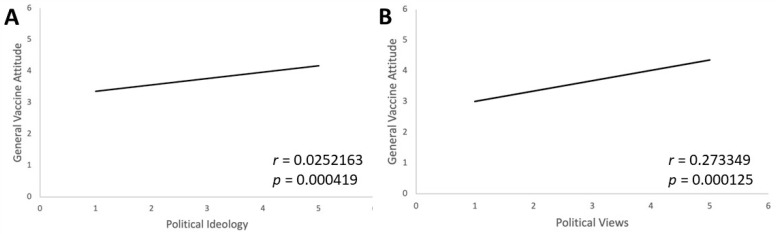
Political ideology and views correlate with intent to vaccinate. Two items related to political ideology were given on the follow-up survey: (**A**) “Please select the opinion that best describes your political ideology” with 5 possible response choices showing if participants are very conservative, somewhat conservative, neither conservative nor liberal, somewhat liberal, or very liberal and (**B**) “How has the COVID-19 pandemic influenced your political views?” with 5 possible response choices, all beginning with “My political views now lean...”, followed by either much more conservatively, a little more conservatively, not changed, a little more liberally, or much more liberally. Each places very conservative at 1 and very liberal at 5. These responses were compared to responses to the question “How has your experience with the COVID-19 global pandemic affected your opinion on vaccinations in general (not including the COVID-19 vaccine)?” with more positive attitudes being in the higher number range. Both liberal political ideology and influence of the pandemic on political views in a more liberal direction were strongly positively correlated to positive general attitudes towards vaccines (both *p* < 0.0005).

Respondents were asked about their political ideology and views to see the correlation between political beliefs and vaccine attitudes. The more liberal-leaning an individual, the higher their number on the political scale. Similarly, the higher an individual’s vaccine attitude, the higher their number was on the general vaccine attitude scale. Figure 6 shows a positive correlation (r = 0.252163) with respondents who selected that they were more liberal, indicating a more positive intent to vaccinate as a result of the pandemic (*p* < 0.001). In addition, respondents who claimed that their political views became much more liberal due to the COVID-19 pandemic had a higher positive attitude towards vaccinations in general compared to respondents whose political views became much more conservative. This highlights that individuals who are more liberal in their political leanings have more positive general vaccination attitudes compared to individuals who lean conservatively. 

### 3.7. Public Safety Measures 

Opinions on mask effectiveness and intent to vaccinate in general were positively correlated (r= 0.365034872, *p* < 0.001), with the belief that masks are effective towards limiting spreading being associated with a greater favorability towards vaccines in general. In addition to mask use, other public safety measures were also positively correlated with vaccine opinions. For example, positive opinions on the effectiveness of public sanitation measures were correlated with better vaccine opinions (r = 0.234594495, *p* < 0.01). Positive opinions on the effectiveness of quarantining were also correlated with more positive vaccine opinions (r = 0.24061, *p* < 0.001). 

A statistically significant difference (*p* < 0.001) between responses about mask effectiveness earlier in the pandemic as opposed to later on was found. Mean values dropped from 4.272 in the initial study to 3.602 in the follow-up study, with fewer people believing that masks were effective in preventing the spread of COVID-19. 

### 3.8. Fertility 

The concern and rumors regarding the COVID-19 vaccine affecting women’s fertility came about after the original survey and therefore, a comparative question was not used in paired data analysis. Respondents to the follow-up survey were asked if the statement ‘The COVID-19 vaccine harms women’s fertility’ was true or false. A total of 9.9% of respondents believed this statement to be true, while 90.1% of respondents believed this statement was false. After performing a Pearson Test, a positive correlation was found between the belief that the COVID-19 vaccine does not cause infertility in women and one’s likelihood to vaccinate against COVID-19 (*p* = 0.00338). 

### 3.9. Primary News Sources 

Respondents were asked about their primary source of information regarding COVID-19. In the survey (Q5.3), respondents were asked to choose between “Your primary doctor”, “CDC, WHO, or local board of health”, “Local news”, “Friends or social media”, “Celebrities/public figures”, “Religious leaders”, “Political leaders”, “Other scientists”, or “Other”. A total of 42.71% of respondents reported that they received COVID-19 information primarily from the CDC, WHO, or a local board of health. A total of 17.19% reported that they received information primarily from “local news”, and 17.71% reported that their primary source of information was “friends or social media”. These results were treated with a healthy degree of skepticism as self-reporting is known to have a significant bias towards ideal behaviors [29]. There was no significant association found between the primary source of information and vaccine attitudes (*p* = 0.67185962). 

To determine which of the variables is most associated with improvement in general vaccine attitudes, we ran a stepwise regression. Out of nine variables (trust in the pharmaceutical industry, trust in healthcare, trust in medicine, trust in government, perceived impact of the COVID-19 pandemic, political views, news and media sources, trust in public measures, and perceived fertility issues), four were determined to be significant. These variables were political ideology, (log worth = 4.254, *p* < 0.0001) trust in healthcare (log worth = 3.870, *p* = 0.0001), trust in medicine (log worth = 2.989, *p* = 0.001), and perceived fertility issues (log worth = 2.264, *p* = 0.00544). 

## 4. Discussion

As we learned in our previous work [9], most people in the United States that we surveyed viewed the COVID-19 vaccine as necessary. This was in large part due to their opinions that the COVID-19 pandemic was a severe problem at the time. There are many factors that contribute to the public’s willingness to receive vaccines in general after experiencing a pandemic of the magnitude we experienced with the COVID-19 virus.

One of the most significant factors in the public’s general vaccine hesitancy post-COVID-19 was trust in the pharmaceutical industry, healthcare, and medicine. While significant positive correlations were found between trust in the pharmaceutical industry, healthcare, and medicine, each with positive general vaccine attitudes, there was no significant correlation found between trust in government and general vaccine attitudes. This finding suggests that if efforts are made to increase trust in the pharmaceutical industry, healthcare, and medicine, then general vaccine attitudes may improve.

During the COVID-19 pandemic, the vaccine became somewhat of a political matter. Our data show that a person’s political beliefs had a significant effect on changes in their general vaccine attitudes due to the COVID-19 pandemic. First, those with more liberal political beliefs were found to be positively correlated with improved vaccine attitudes. Next, political beliefs, in general, shifted more liberal through the pandemic, and those who became more liberal also had a better opinion of vaccines in general (Figure 6). Taken together, these two findings suggest that experiencing the COVID-19 pandemic increased positive vaccine attitudes through the mediating factor of political beliefs. This is in concordance with other work that has found the right-wing populist rhetoric of political figures such as Donald Trump and Jair Bolsonaro likely impacted the views of their followers towards vaccines and the pandemic [30].

Another significant finding is that those who believed in and participated in public safety measures and wore masks during the COVID-19 pandemic had a more positive general vaccine attitude than those who did not. However, by the end of our study, we found a drop in belief in mask effectiveness compared to the beginning of the pandemic, which may be due to the large influx of contradictory information both on social media and in news outlets throughout the COVID-19 pandemic. 

As the COVID-19 vaccines became available to the public, concerns arose about the long-term effects of the vaccines, specifically the effects the vaccine had on fertility. Those who trusted that the various COVID-19 vaccines did not affect fertility were significantly more likely to get fully vaccinated against COVID-19. 

We drew no significant conclusions on the effect of news sources on intent to vaccinate against COVID-19. This could be due to a self-reporting bias present from our respondents [29]. In future studies, it would be wise to craft survey design and analysis in a way that will successfully capture the effect of news sources on vaccine hesitancy as this is a significant public health concern. Although conspiracy theories certainly had an impact on vaccine attitudes and the COVID-19 response, these may be unlikely to persist [31].

Overall, the respondents in our survey had an increased positive attitude towards vaccines after experiencing a pandemic. While a slight majority reported no change in general vaccine attitudes, a high percentage–43.75%—reported that they were much more likely to vaccinate themselves or their children after experiencing the COVID-19 pandemic. 

### Limitations

The number of people who completed both the initial survey and the follow-up survey is relatively small. Although these results may not be able to be extrapolated to the entire population, they provide a look into a specific subset of people who were highly affected by the pandemic (classes cancelled, housing disrupted, etc.) and who are at an important time for decision making in their lives. 

Since this work was done at universities in Utah, the population is somewhat homogenous, as may be seen by the lack of ethnic diversity. This should be taken into consideration when generalizing the results. 

## 5. Conclusions

Through this study, we found that there were increased positive attitudes among college students towards vaccination after experiencing the COVID-19 pandemic. We also found that higher trust in the pharmaceutical industry, healthcare and medicine, and public safety measures like mask use and public sanitation was positively correlated with overall positive vaccine attitudes. Notably, we found a difference in responses regarding mask effectiveness between the two surveys with fewer people thinking masks are effective now as compared to the beginning of the pandemic. We found that those who hold more liberal political views are more likely to have more positive vaccine attitudes (Figure 6). We also predicted that trust in government and self-reported primary news sources have an effect on general vaccine attitudes. However, we found no significant correlation between either of those variables and general vaccine attitudes. 

## Figures and Tables

**Table 1:** Respondent demographics.

	Number	Percentage
Age		
Less than 18	0	0
18–25	184	95.8
26–35	3	1.56
36–45	3	1.56
46–55	2	1.04
Over 55	0	0
Race/Ethnicity		
American Indian or Alaskan Native	0	0
Asian	9	4.69
Black or African American	0	0
Hispanic or Latino	5	2.60
Native Hawaiian or Pacific Islander	2	1.04
White	175	91.14
Other	0	0
Prefer not to answer	1	0.52
Sex		
Male	99	51.56
Female	92	47.92
Non-Binary/Third Gender	1	0.52
Prefer to self-describe	0	0
Prefer not to answer	0	0
Number of children		
0	184	95.34
1	6	3.11
2	0	0
More than 2	3	1.56

## Data Availability

The survey is available in the Supplemental Files. Aggregate data is available upon request.

## Supplementary Materials

The following supporting information can be downloaded at: https://www.mdpi.com/article/10.3390/vaccines11071132/s1, Supplementary File S1: Initial Survey; Supplementary File S2: Follow-up survey.
