# Supplementary material for: Decrease in Overall Vaccine Hesitancy in College Students during the COVID-19 Pandemic
Source: Vaccines (Basel). 2023 Jun 21;11(7):1132. doi: 10.3390/vaccines11071132 (PMC10384532; doi:10.3390/vaccines11071132)
Supplement: Supplementary file 1 [file vaccines-11-01132-s001.zip › Supplementary File S1-Follow up survey.pdf]

Part 2A

Thank you for taking this follow-up survey. This survey is a follow-up survey to a survey that you completed last year about COVID-19 and vaccinations.

Please enter your email.

Please select ALL of the following statements that apply to your experience with COVID-19:

- I have tested positive for a COVID-19 infection
- An immediate family member has tested positive for COVID-19 infection
- An extended family member has tested positive for COVID-19 infection
- A roommate or friend who lives with me has tested positive for COVID-19 infection
- A friend who does not live with me has tested positive for COVID-19 infection
- A coworker has tested positive for COVID-19 infection
- A distant friend or a friend of a friend has tested positive for COVID-19 infection
- I do not know anyone who has tested positive for COVID-19 infection

How severe was *your COVID-19 infection*?

No symptoms/mild symptoms

Moderate symptoms but health care providers were not contacted

Moderate symptoms and health care providers were contacted

Severe symptoms/hospitalization

How severe were the symptoms of the COVID-19 infection of *your immediate family member?*

No symptoms/mild symptoms

Moderate symptoms but health care providers were not contacted

Moderate symptoms and health care providers were contacted

Severe symptoms/hospitalization

Death

How severe were the symptoms of the COVID-19 infection of *your extended family member?*

No symptoms/mild symptoms

Moderate symptoms but health care providers were not contacted

Moderate symptoms and health care providers were contacted

Severe symptoms/hospitalization

Death

How severe were the symptoms of the COVID-19 infection of *your friend who does not live with you?*

No symptoms/mild symptoms

Moderate symptoms but health care providers were not contacted

Moderate symptoms and health care providers were contacted

Severe symptoms/hospitalization

Death

How severe were the symptoms of the COVID-19 infection of *the roommate or friend you live with?*

No symptoms/mild symptoms

Moderate symptoms but health care providers were not contacted

Moderate symptoms and health care providers were contacted

Severe symptoms/hospitalization

Death

How severe were the symptoms of the COVID-19 infection of *your coworker?*

No symptoms/mild symptoms

Moderate symptoms but health care providers were not contacted

Moderate symptoms and health care providers were contacted

Severe symptoms/hospitalization

Death

How severe were the symptoms of the COVID-19 infection of *your distant friend or friend of a friend?*

No symptoms/mild symptoms

Moderate symptoms but health care providers were not contacted

Moderate symptoms and health care providers were contacted

Severe symptoms/hospitalization

Death

Part 2B

How has your experience with the COVID-19 global pandemic affected your opinion on vaccinations in general (not including the COVID-19 vaccine)?

I am much more likely to vaccinate myself/my children

I am more likely to vaccinate myself/my children

My opinion on vaccinations has not changed

I am less likely to vaccinate myself/my children

I am much less likely to vaccinate myself/my children

What is your current employment status? Check ALL that apply.

Working full time for pay (please specifiy number of hours per week)

Working part time for pay (please specify number of hours per week)

Not currently employed, looking for work

Retired

Homemaker

Disabled (not working because of a permanent or temporary disability)

Other (please specify)

How has the COVID-19 pandemic affected your employment/income?

Severely

Moderately

Mildly

Not at all

Improved

How has the COVID-19 pandemic affected your mental health? My mental health was ______ during the pandemic, compared to before the pandemic.

Much better

Somewhat better

About the same

Somewhat worse

Much worse

How has the COVID-19 pandemic affected your ability to carry out your normal activities?

Dramatic restrictions

Moderate restrictions

No restrictions

Moderately easier

Much easier

Part 2C

How closely did you follow news regarding COVID-19 in the beginning of the pandemic?

Very closely

Somewhat closely

An average amount

Not very closely

Not at all

How closely do you follow news regarding COVID-19 right now?

Very closely

Somewhat closely

An average amount

Not very closely

Not at all

What is your primary source of information regarding COVID-19?

Your primary doctor

CDC (Center for Disease Control and Prevention), WHO (World Health Organization) or local Board of Health

Other scientists

Local news

Friends or Social Media

Celebrities/public figures

Religious leaders

Political leaders

Other (Please specify)

Part 2C (second half)

Please indicate whether the following statements are true or false:

Ingesting Ivermectin as a treatment for COVID-19 will reduce symptoms and prevent hospitilization.

True

False

Drinking your own urine can act as an antidote/cure for COVID-19 infection.

True

False

On average it takes 5–6 days from when someone is infected with COVID-19 for symptoms to show, however it can take up to 14 days.

True

False

Using Hydroxychloroquine for hospitalized patients with COVID-19 was both a safe and effective treatment for COVID-19.

True

False

Once you contract COVID-19, the virus can never be eliminated from your body.

True

False

Symptoms of COVID-19 can include sore throat, diarrhea, and conjunctivitis (eye infection).

True

False

The COVID-19 vaccine harms women's fertility.

True

False

Most people who contract COVID-19 will recover from it.

True

False

Part 2D

I am current on the vaccinations recommended by my primary care physician.

I am current on all

I am current on most

I am current on some

I am not current on any

I am uncertain

How important is it for you to get the flu vaccine every year?

Very important

Important

Somewhat important

Not very important

Not at all important

How important is it for you to get the COVID-19 vaccine if you have already been infected with the virus?

Very important

Important

Somewhat important

Not very important

Not at all important

Part 2E

What is your COVID-19 vaccination status?

Fully vaccinated including booster against COVID-19

Fully vaccinated but no COVID-19 booster

First COVID-19 vaccine only

Not vaccinated against COVID-19

Which of these statements most closely resembles your reason for choosing to vaccinate yourself:

I believe the vaccine is safe

I believe the vaccine is effective

I trust the source(s) that encouraged me to get the vaccine

I do believe in all vaccines, and my reason is not any different for a new COVID-19 vaccine

I got vaccinated for travel purposes

I got vaccinated for work purposes

I got vaccinated for social purposes

Other

Which of these statements most closely resembles your reason for choosing to not vaccinate yourself:

I do not believe the vaccine is safe

I do not believe the vaccine is effective

I do not trust the source(s) that encouraged me to get the vaccine

I do not believe in any vaccines, and my reason is not any different for a new COVID-19 vaccine

A source that I trust encouraged me to NOT get the vaccine

Other

Which brand of COVID-19 vaccination did you receive? (check all that apply)

Pfizer-BioNTech

Moderna

Astra-Zeneca

Johnson & Johnson's Janssen

If you have children, have you/are you likely to vaccinate them against COVID-19?

I do have children and I would vaccinate them

I do have children and I would not vaccinate them

I do not have children

Which of these statements most closely resembles your reason for choosing to vaccinate your children:

I believe the vaccine is safe for children

I believe the vaccine is effective

I trust the source(s) that encouraged me to give my child the vaccine

I believe in all vaccines, and my reason is not any different for a new COVID-19 vaccine

Other

Which of these statements most closely resembles your reason for choosing to not vaccinate your children:

I do not believe the vaccine is safe for children

I do not believe the vaccine is effective

I do not trust the source(s) that encouraged me to give my child the vaccine

I do not believe in any vaccines, and my reason is not any different for a new COVID-19 vaccine

I am indifferent to having my child receive the vaccine, but probably will not end up having my child receive it

Other

Once the COVID-19 vaccine became available to you, how much of a priority was it for you to get vaccinated?

Very low priority

Somewhat of a low priority

Not a high priority nor a low priority

Somewhat of a high priority

Very high priority

How helpful do you think the COVID-19 vaccines were in controlling the pandemic?

Very unhelpful

Somewhat unhelpful

Neither helpful nor unhelpful

Somewhat helpful

Very helpful

Part 2F

Vaccines are important for the prevention of serious diseases.

Strongly agree

Agree

Uncertain

Disagree

Strongly disagree

The administration of more than one vaccine at the same time can be unsafe for my child’s health.

Strongly agree

Agree

Uncertain

Disagree

Strongly disagree

The side effects of most vaccines outweigh the benefits

Strongly agree

Agree

Uncertain

Disagree

Strongly disagree

I worry that the rushed pace of testing for a new COVID-19 vaccine will fail to detect potential side effects or dangers.

Strongly agree

Agree

Uncertain

Disagree

Strongly disagree

My children are current on which recommended vaccines (or, if I don't have children, I would keep my children current on which recommended vaccines)?

All recommended vaccines

Most recommended vaccines

Some recommended vaccines

Uncertain

My children are not/would not be vaccinated

Part 2G

Please answer the following questions in your own words: The biggest fear I have about a COVID-19 vaccine is...

Rank how much you agree with the following statements regarding the COVID-19 pandemic?

Masks were effective in protecting against COVID-19.

Strongly disagree

Disagree

Uncertain

Agree

Strongly agree

Social distancing was effective in protecting against COVID-19.

Strongly disagree

Disagree

Uncertain

Agree

Strongly agree

Public sanitation measures (such as hand-washing, hand sanitizing, cleaning public areas, etc.) were effective in protecting against COVID-19.

Strongly disagree

Disagree

Uncertain

Agree

Strongly agree

Quarantine mandates were effective in preventing the spread of COVID-19.

Strongly disagree

Disagree

Uncertain

Agree

Strongly agree

How much of a problem was COVID-19 in America?

Not a problem at all

Insignificant compared to other problems

Somewhat of a problem

A severe problem, more important than most other issues

The most important problem America faced/is facing right now

part 1: Demographic information

These questions are helpful to the researchers in comparing similar groups of people and to help us understand the results of the research better. Although these may be personal questions, your name and specific information will not be associated with the survey. Please select the best answer for each question.

Marital Staus

Single

Married

Divorced

Widow/widower

Number of children

0

1

2

More than 2

Level of education

Have not finished high school

Finished high school

Some college

Associates degree

Bachelors degree

Masters degree

Doctoral degree (e.g., MD,DDS,PhD)

Political affiliation

Democrat

Republican

Independent

Please select the opinion that best describes your political ideology

Very conservative

Somewhat conservative

Neither conservative not liberal

Somewhat liberal

Very liberal

How has the COVID-19 pandemic influenced your political views?

My political views now lean much more conservatively

My political views now lean a little more conservatively

My political views have not changed

My political views now lean a little more liberally

My political views now lean much more liberally

Block 9

During the pandemic, my trust in the government has...

Decreased greatly

Decreased somewhat

Did not change

Increased somewhat

Increased greatly

During the pandemic, my trust in the pharmaceutical industry has...

Decreased greatly

Decreased somewhat

Did not change

Increased somewhat

Increased greatly

During the pandemic, my trust in medicine has...

Decreased greatly

Decreased somewhat

Did not change

Increased somewhat

Increased greatly

During the pandemic, my trust in healthcare has...

Decreased greatly

Decreased somewhat

Did not change

Increased somewhat

Increased greatly

Powered by Qualtrics
