# Supplementary material for: Decrease in Overall Vaccine Hesitancy in College Students during the COVID-19 Pandemic
Source: Vaccines (Basel). 2023 Jun 21;11(7):1132. doi: 10.3390/vaccines11071132 (PMC10384532; doi:10.3390/vaccines11071132)
Supplement: Supplementary file 1 [file vaccines-11-01132-s001.zip › Supplementary File S2-Initial Survey.pdf]

BYU
BRIGHAM YOUNG
UNIVERSITY

Consent

Consent to be a Research Subject

Title of the Research Study: Impact of the COVID-19 pandemic on overall vaccine attitudes

Principal Investigator: Brian Poole

IRB ID#:

-

Introduction

This research study is being conducted by **Brian Poole** at Brigham Young University to determine the effects of the COVID-19 pandemic on people's attitudes about vaccines. You were invited to participate because **you are a university student in Utah.**

Procedures

If you agree to participate in this research study, the following will occur:

· You will complete this survey that will begin after you select "accept." The survey will take approximately 20 minutes.

· If you choose to provide your email address, the researcher will contact you in approximately one year to measure any change in opinions.

· total time commitment will be 40 minutes

Risks/Discomforts

The risks of this study are that your answers may be revealed somehow, and you may feel embarrassment at having your answers revealed. If your answers are associated with you and revealed to the professors who are teaching your classes, they may alter their opinion of you. These risks are considered minimal and unlikely.

To protect against release of your information, your answers will be stored on servers maintained by the Qualtrics Corporation. Once downloaded, your information will be de-identified before being shown to any professor or anyone associated with a class you are in. All information will be kept in password-protected files on password-protected computers kept in a locked room.

Benefits

There is no direct benefit to you as a research subject. Benefits to society may include a greater understanding of how people make decisions about vaccines.

Confidentiality

All data will be stored on commercially encrypted servers. Once downloaded, data will be de-identified, with your name and email replaced by a code, by research assistants and stored on password-protected computers. The de-identified data will be kept for future studies. If you choose to participate in the second part of the study, you will receive a second email invitation after approximately a year, and any data you provide will be treated in the same way. If you choose to provide your name and class to receive extra credit, your name will be included as part of a list of names of those who participated, with no other information associated with your name.

Data Sharing

We will keep the information we collect about you during this research study for analysis and for potential use in future research projects. Your name and other information that can directly identify you will be stored securely and separately from the rest of the research information we collect from you.

If you agree, the researchers plan to contact you again as part of this research study.

De-identified data from this study may be shared with the research community, with journals in which study results are published, and with databases and data repositories used for research. We will remove or code any personal information that could directly identify you before the study data are shared. Despite these measures, we cannot guarantee anonymity of your personal data.

Compensation

You will receive extra credit for your completion of the initial survey, not to exceed 1% of the total class credit. Extra credit will not be prorated or withdrawn for refusing to participate in the second survey to follow in a year. Additional extra credit will not be given for the second survey.

Participation

Participation in this research study is voluntary. You have the right to withdraw at any time or refuse to participate entirely without jeopardy to your class status, grade, or standing with the university. An alternate extra credit possibility will be provided if you choose not to participate.

Questions about the Research

If you have questions, concerns, or complaints, you can contact the Principal Investigator **Brian Poole, at 801-422-8092.**

Questions about Your Rights as Research Participants

If you have questions regarding your rights as a research participant contact Human Research Protections Manager by phone at (801) 422-1461; or by email: irb@byu.edu.

Statement of Consent
I have read, understood, and received a copy of the above consent and desire of my own free will to participate in this study. Please type your name as an electronic signature

Date

I consent to be contacted in approximately one year for a follow-up survey

Yes

No

Please provide your email address so we may contact you for the follow-up survey

Demographic Information

These questions are helpful to researchers in comparing similar groups of people and to better understand survey results for research. Although these questions are of a personal nature your name and any identifying information will not be associated with the survey results. Please select the best answer for each question.

Age:

18-25

26-35

36-45

46-55

Over 55

Race:

American Indian or Alaskan Native

Asian

Black or African American

Hispanic or Latino

Native Hawaiian or or Pacific Islander

White

Prefer not to answer

Other (Please specify)

Sex:

Male

Female

Non-binary/third gender

Prefer not to answer

Prefer to self-describe

Martial History:

Single

Married

Divorced

Widow/widower

Number of children:

0

1

2

More then 2

Education:

Have not finished high school

Finished high school

Some college

Associate's Degree

Bachelor's Degree

Post-baccalaureate/professional degree (e.g. Masters, MD, DDS, PhD)

Political ideology:

Far Left Liberal

Moderate Liberal

Moderate Conservatice

Far Right Conservative

Other (please specify)

General Religious Affiliation:

Buddhism

Christianity

Hinduism

Islam

Judaism

Other

No Religious Affiliation

Specific Religious Affiliation (Buddhism)

Theravāda (Teaching of the Elders, Southern Buddhism)

Mahāyāna (Great vehicle, East Asian Buddhism)

Vajrayāna (Tantric/Esoteric Buddhism)

Navayāna

Other (please specify)

Specific Religious Affiliation (Christianity)

Anglican/Episcopalian

Baptist

Catholic

Christian (non-denominational)

Church of Christ/Disciples of Christ

Congregational

Jehovah's Witness

LDS (Mormon)

Lutheran

Methodist/Wesleyan

Orthodox (Eastern)

Pentecostal/Charismatic

Protestant (Other)

Reformed/Presbyterian

Seventh-day Adventist

Other (please specify)

Specific Religious Orientation (Hinduism)

Vaishnavism

Shaivism

Shaktism

Smartism

Other (please specify)

Specific Religious Affiliation (Islam)

Shia

Sunni

Sufi

Other (please specify)

Specific Religious Affiliation (Judaism)

Conservative

Orthodox

Reformed

Other (please specify)

Specific Religious Affiliation (Other)

Jainism

Shintoism

Rastafarianism

Sikhism

Confucianism

Zoroastrianism

Pagan/neo-pagan

Traditional African

African Diaspora

Indigenous American

Aboriginal

Folk Religions (please specify)

Other (please specify)

Specific Religious Affiliation (No Religious Affiliation)

Agnostic

Atheist

Spiritual, but no specific affiliation

Other (please specify)

Physical health effects

What was the most severe outcome of COVID for someone you know personally?

No symptoms/mild symptoms but positive test results

Moderate symptoms but health care providers were not contacted

Moderate symptoms and health care providers were contacted

Severe symptoms/hospitalization

Death

Please select all of the following statements that apply to your experience with COVID-19

I have tested positive for a COVID-19 infection

An immediate family member has tested positive for COVID-19

An extended family member has tested positive for COVID-19

A friend that does not live with me has tested positive for COVID-19

Someone I live with has tested positive for COVID-19

A coworker has tested positive for a COVID-19 infection

A distant friend or a relative's friend tested positive for COVID-19

How severe was *your Covid-19 infection?*

No symptoms/mild symptoms

Moderate symptoms but health care providers were not contacted

Moderate symptoms and health care providers were contacted

Severe symptoms/hospitalization

How severe was the *Covid-19 infection of your immediate family member?*

No symptoms/mild symptoms

Moderate symptoms but health care providers were not contacted

Moderate symptoms and health care providers were contacted

Severe symptoms/hospitalization

Death

How severe was the Covid-19 infection of your extended family member?

No symptoms/mild symptoms

Moderate symptoms but health care providers were not contacted

Moderate symptoms and health care providers were contacted

Severe symptoms/hospitalization

Death

How severe was the COVID-19 infection of your friend that does not live with you?

No symptoms/mild symptoms

Moderate symptoms but health care providers were not contacted

Moderate symptoms and health care providers were contacted

Severe symptoms/hospitalization

Death

How severe was the COVID-19 infection of the individual that lives with you?

No symptoms/mild symptoms

Moderate symptoms but health care providers were not contacted

Moderate symptoms and health care providers were contacted

Severe symptoms/hospitalization

Death

How severe was the COVID-19 infection of your coworker?

No symptoms/mild symptoms

Moderate symptoms but health care providers were not contacted

Moderate symptoms and health care providers were contacted

Severe symptoms/hospitalization

Death

How severe was the COVID-19 infection of your distant friend or relative’s friend?

No symptoms/mild symptoms

Moderate symptoms but health care providers were not contacted

Moderate symptoms and health care providers were contacted

Severe symptoms/hospitalization

Death

Mental health effects

Did you experience any new or worsening mental health issues due to your experience durring the COVID-19 pandemic?

Yes, dramatic changes

Yes, slight changes

No changes

Did you experience new or worsening anxiety issues due to COVID-19 pandemic?

Yes

No

Did you experience new or worsening depression issues due to COVID-19 pandemic?

Yes

No

Did you need to start or increase medications to combat your anxiety and/or depression during the COVID-19 pandemic?

Yes

No

Did you feel more lonely during the COVID-19 pandemic then you felt beforehand?

Yes

No

General effects of pandemic

How has the COVID-19 pandemic changed your attitude to infectious diseases in America

It has made me much more concerned about infections diseases

It has made me somewhat more concerned about infectious diseases

It has not changed my attitude to infectious diseases

It has made me somewhat less concerned about infectious diseases

It had made me much less concerned about infectious diseases

The COVID-19 pandemic did not change my attitude to infectious diseased because:

I was already very concerned about infectious diseases and the pandemic did not increase my concern

I was already very unconcerned about infectious diseases and the pandemic did not increase my concern

Other (please specify)

How much did the COVID-19 pandemic affect your life?

Severely impacted my life

Moderatly impacted my life

No impact on my life

How did the COVID-19 pandemic affect your income?

Slight income increase

Decreased income

Lost Job

Furloughed

Accepted a new Job

To what extent did the COVID-19 pandemic limit your activities?

Severely limited my activities

Moderately limited my activities

Had no impact on my activities

What is the most memorable example of how the COVID-19 pandemic limited your activities?

How has your experience with the COVID-19 Pandemic affect your opinion on vaccinations in general?

I am much more likely to vaccinate myself or my children

I am more likely to vaccinate myself or my children

My opinion on vaccinations has not changed

I am less likely to vaccinate myself or my children

I am much less likely to vaccinate myself or my children

Why has the pandemic not affected your opinion on vaccinations in general?

The Pandemic had little effect on my opinion because I already feel very positively toward vaccination in general

The Pandemic had little effect on my opinion because I already feel very negatively toward vaccination in general

Other (please specify)

Indicate how you feel about the following statement: People my age are unlikely to be seriously affected by the virus

Strongly agree

Somewhat agree

Uncertain

Somewhat disagree

Strongly disagree

Please indicate how strongly you agree or disagree with each of the following statements:

	Strongly agree	Somewhat agree	uncertain	Somewhat disagree	Strongly disagree
When worn correctly, masks prevent the spread of COVID-19.	○	○	○	○	○
False positives are common with the current COVID-19 tests.	○	○	○	○	○
It is possible to spread COVID-19 up to two days before you start showing symptoms.	○	○	○	○	○
The current COVID-19 testing methods are unreliable/inaccurate	○	○	○	○	○

Vaccine History

Are you planning on getting a Flu (influenza) vaccine this year?

Yes

No

I already revived a Flu vaccine this year

In which of the previous years did you receive a flu vaccine?

2020

2019

2018

2017

Were you more inclined to get a flu vaccine in 2020 then in previous year due to the COVID-19 pandemic?

Yes

No

Have you ever received a vaccine in order to travel to another country?

Yes

No

Have you received a COVID-19 vaccine?

Yes

No

When did you receive the COVID-19 vaccine? (MM/YYYY)

Indicate the primary reason why you have not received a COVID-19 vaccine.

The vaccine is not available to me yet

The vaccine is too expensive

I do not feel that that the vaccine has undergone enough testing to ensure it's safety

I do not feel that COVID-19 is dangerous enough to warrant getting vaccinated

Other (please specify)

Vaccine and general knowledge

Please indicate how strongly you agree or disagree with each of the following statements about vaccines (Note: these statements refer to vaccines in general rather then to a specific vaccine)

	Strongly agree	Somewhat agree	Neither agree nor disagree	Somewhat disagree	Strongly disagree
Vaccines are more helpful than harmful	○	○	○	○	○
Vaccines often have severe side effects	○	○	○	○	○
Vaccines cause autism	○	○	○	○	○
I am likely to fully vaccinate my children/ I have fully vaccinated my children	○	○	○	○	○
I am worried that a vaccine will give me the disease that it is designed to prevent	○	○	○	○	○
I would rather build immunity by exposure to an infected individual than receive a vaccine.	○	○	○	○	○
I am worried about the financial cost of vaccines.	○	○	○	○	○
The side effects of a vaccine are likely to be worse then the effects of the actual disease	○	○	○	○	○
I would feel more comfortable getting a vaccine if I was provided a comprehensive list of ingredients	○	○	○	○	○
Not everyone who is eligible for a vaccine needs to receive it because herd	○	○	○	○	○

Please indicate how strongly you agree or disagree with each of the following statements about General health care

	Strongly agree	somewhat agree	Unsure	Somewhat disagree	Strongly disagree
Treatments for autoimmune diseases are more helpful than harmful	○	○	○	○	○
There are effective treatments for autoimmune diseases	○	○	○	○	○
Exercise is the best treatment for autoimmune diseases	○	○	○	○	○
People with autoimmune diseases suffer considerably	○	○	○	○	○
Doctors (in general) care about their patients' health just as much or more than their patient's do	○	○	○	○	○
Doctors sometimes do not pay attention to or disregard what their patients are telling them	○	○	○	○	○
Doctors are competent, careful and well trained	○	○	○	○	○
Prescribed treatments are more beneficial than harmful	○	○	○	○	○

Vaccine information sources

Which of the following information sources has influenced your attitude toward vaccines the most during this pandemic?

Blogs or other social media

CDC (Centers for Disease Control and Prevention)

Comments by political figures

Discussion with friends

Local News

Religious Counsel

WHO (World Health Organization)

Please rank the following sources from most reliable/accurate to least reliable/accurate

Blogs or other social media

CDC (Centers for Disease Control and Prevention)

Comments by political figures

Discussion with friends

Local News

Religious Counsel

WHO (World Health Organization)

How closely do you follow the news regarding COVID-19?

Very closely

Somewhat closely

Not very closely

Not at all

How are vaccines generally portrayed in the information sources that you normally watch, read, listen to?

Strongly encouraged

Encouraged

Neutral/not portrayed

Discouraged

Strongly discouraged

Since the Pandemic I have been become more careful about which information sources I trust

Strongly agree

Somewhat agree

Neither agree nor disagree

Somewhat disagree

Strongly disagree

Powered by Qualtrics
